# Seroprevalence of Measles Antibodies Among Migrant Populations: A Systematic Review and Meta-Analysis

**DOI:** 10.7759/cureus.74243

**Published:** 2024-11-22

**Authors:** Somen K Pradhan, Ashutosh Panda, Ipsita Debata, Prem S Panda

**Affiliations:** 1 Department of Community Medicine, Maharaja Krishna Chandra Gajapati Medical College & Hospital, Brahmapur, IND; 2 Department of Community Medicine, Kalinga Institute of Medical Sciences, Bhubaneswar, IND

**Keywords:** herd immunity, measles, migrant population, refugee, seroprevalence, vaccination

## Abstract

Incomplete or interrupted vaccination schedules put migrant communities at higher risk for measles, which remains a serious public health concern. The objective of this systematic review was to evaluate the pooled seroprevalence of measles antibodies among migrant groups globally and offer data to guide public health initiatives. Our literature search included PubMed, Scopus, and Embase databases, covering publications from 1990 to 2023, and was systematically refined using specific inclusion and exclusion criteria. Only observational studies documenting measles antibody seroprevalence among defined migrant groups were included to ensure relevance and quality in addressing the study’s objective. Meta-analytical techniques such as random-effects models were employed to assess pooled seroprevalence. The meta-analysis comprised 36 studies with 42,972 participants in total. Among migrant communities, the overall pooled seroprevalence of measles antibodies was 83% (95%CI: 80%-87%). Studies showed significant heterogeneity (I^2^ = 98%, p < 0.01). According to meta-regression analysis, measles seroprevalence has been gradually increasing in more recent research, and time (year) was a significant predictor of seroprevalence variability (p < 0.05). Measles seroprevalence in migratory communities is still below the threshold for herd immunity, especially in susceptible populations like children and refugees. To close these immunity gaps and stop future outbreaks in host nations, focused public health interventions such as catch-up vaccination programs are desperately needed.

## Introduction and background

Despite international efforts to eradicate the disease through vaccination campaigns, measles continues to pose a serious threat to public health. Although measles vaccine is thought to have avoided nearly 57 million deaths between 2000 and 2022, outbreaks still happen all over the world, especially in areas with low vaccination rates, according to the World Health Organization (WHO) [1,2].

An important turning point in the battle against measles was the development of a highly successful vaccine in the latter half of the 20th century, which led to a sharp decline in both cases and fatalities. Due to regular childhood vaccinations and additional vaccination campaigns, measles rates decreased worldwide and the disease was deemed eradicated in many wealthy nations. A key component of these initiatives is the measles, mumps, and rubella (MMR) vaccination, which offers 93% protection with a single dose and 97% protection with two doses. Mass immunization efforts in conjunction with routine vaccination have been shown to be successful in lowering measles-related mortality and averting widespread outbreaks [3]. However, measles has returned to several regions of the world due to irregularities in immunization programs and gaps in vaccination coverage. Measles epidemics have been especially noticeable among vulnerable groups, including refugees and migrants. Due to difficulties maintaining regular immunizations, lack of vaccination records, and interrupted healthcare access, these groups are more vulnerable to diseases that can be prevented by vaccination. Due to poor living conditions, interrupted immunization schedules, and obstacles to healthcare access, vaccine-preventable illnesses like measles disproportionately afflict migrant communities, including refugees, asylum seekers, and other mobile population groups [4,5].

Measles outbreaks in Europe, Asia, and Africa in recent years have frequently been connected to population shifts, highlighting the significance of closing immunity gaps among migrants [6]. Many migrants arrive in host nations with incomplete or unclear immunization histories, and they are frequently left out of standard vaccination efforts. Their vulnerability is exacerbated by elements like restricted outreach to migrant groups, lack of integration into healthcare institutions, and socioeconomic instability [7]. Significant differences in vaccination coverage still exist, especially among high-risk groups like migrants, despite the Global Vaccine Action Plan (GVAP) 2011-2020's goal of eradicating measles in at least five WHO regions [8].

Seroprevalence studies are essential for assessing population-level immunity and locating vaccination coverage gaps because they measure the presence of antibodies in a population [9]. Public health officials can use the useful information from these studies to guide disease surveillance initiatives and focused immunization campaigns [10]. Seroprevalence investigations can shed light on the immunological vulnerabilities of migrants, providing information about the efficacy of prior immunization campaigns and the necessity of catch-up immunizations [11].

This systematic review and meta-analysis aims to assess measles antibody levels in migrant populations across different regions to identify immunity gaps and support targeted public health actions. We want to determine the factors that influence changes in measles seroprevalence and estimate the overall immunity levels in migrant populations by combining data from several studies. The results of this meta-analysis can be used to help develop public health strategies that can close immunization gaps and stop measles outbreaks in migrant groups.

## Review

Materials and methods

Information Sources and Search Strategy

This systematic review and meta-analysis was carried out in accordance with the Cochrane Collaboration's recommendations for assessing the seroprevalence of measles in migrant communities worldwide. The Preferred Reporting Items for Systematic Review and Meta-Analyses (PRISMA) standards were followed in the conduct and reporting of the systematic review [12]. The review protocol was registered in the International Prospective Register of Systematic Reviews (PROSPERO) (Registration ID: CRD42023448934). Using no initial language constraints, the PubMed, Scopus, and Embase databases were searched for articles published between January 1, 1990, and December 31, 2023, that reported the seroprevalence of measles infection and immunity in immigrants and refugees. We used a search strategy related to migrants (i.e., “Immigrants” OR “Emigrants” OR “Refugees” OR “foreign born” OR “foreigner” OR “migrants” OR “displaced population” OR “evacuated” OR “internationally adopt*”); AND Measles (i.e., “Measles”); AND seroprevalence (i.e., “Seroepidemiological studies” OR “Seroprevalen*” OR “Seroprotect*” OR “serosurvey” OR “serosurveillance” OR “seroepidemiology” OR “serologic test*”).

Study Selection

We included observational research that documented the seroprevalence of measles IgG serum antibodies among migrants of all ages and from any geographic location.

Inclusion criteria were: (a) Studies that specifically focused on migrant populations, including refugees, immigrants, asylum seekers, international adoptees, seasonal labor migrants, and undocumented migrants, (b) Studies reporting seroprevalence of measles IgG antibodies based on serological testing, allowing for quantitative analysis of immunity levels, (c) Observational studies (i.e. cross-sectional, descriptive and analytical studies, cohort studies), and (d) Studies published in 1990-2023. Exclusion criteria were: (a) Studies lacking clear data on measles seroprevalence rates or those where seroprevalence cannot be isolated for migrant populations specifically, (b) Non-observational study designs such as case reports, case series, opinion pieces, editorials, reviews, and letters to the editor, as they do not provide original data for meta-analysis, (c) Grey literature, unpublished data, and non-peer-reviewed sources (i.e. dissertations or preprints or conference abstracts), and (d) Articles only available in languages other than English.

Data Extraction and Quality Assessment

Covidence software (Melbourne, Australia) was used to import all identified records for filtering and deduplication. A full-text review of possibly suitable papers was conducted after two independent reviewers (AP and SKP) separately examined the abstracts and titles. A third reviewer (PSP) was consulted to settle disagreements. Two authors (SKP and ID) used a standardized extraction form in Microsoft Excel (version 2021; Microsoft Corporation, Redmond, Washington, United States) to extract the data. Study parameters (e.g., author, year, location), population demographics, sample size, and measles seroprevalence were among the extracted data. We used the National Heart, Lung, and Blood Institute's (NHLBI) tool to evaluate the quality of the study [13].

Statistical Analysis

The included papers were qualitatively synthesized to characterize study features and seroprevalence trends in various immigrant groups. One author (PSP) entered the data for the meta-analysis into Microsoft Excel, while another author (ID) confirmed the data. In order to offer a summary measure across the included studies, the pooled estimate of the seroprevalence was computed using a meta-analytical approach for quantitative synthesis (meta-analysis). The I^2^ statistic was used to evaluate heterogeneity. For the meta-analysis, we used R software (version 4.4.1; R Foundation for Statistical Computing, Vienna, Austria) [14]. The expected variance in the populations and methodologies of the individual studies led to the selection of a random-effects model. A forest plot was developed to visually represent each study's effect sizes and associated confidence intervals. Publication bias was assessed using both Egger's and Begg's tests, with significance set at p < 0.05. A random-effects meta-regression model was used to examine the effect of time (year) on logit-transformed measles seroprevalence.

Results

Study Selection and Characteristics

**Figure 1:**
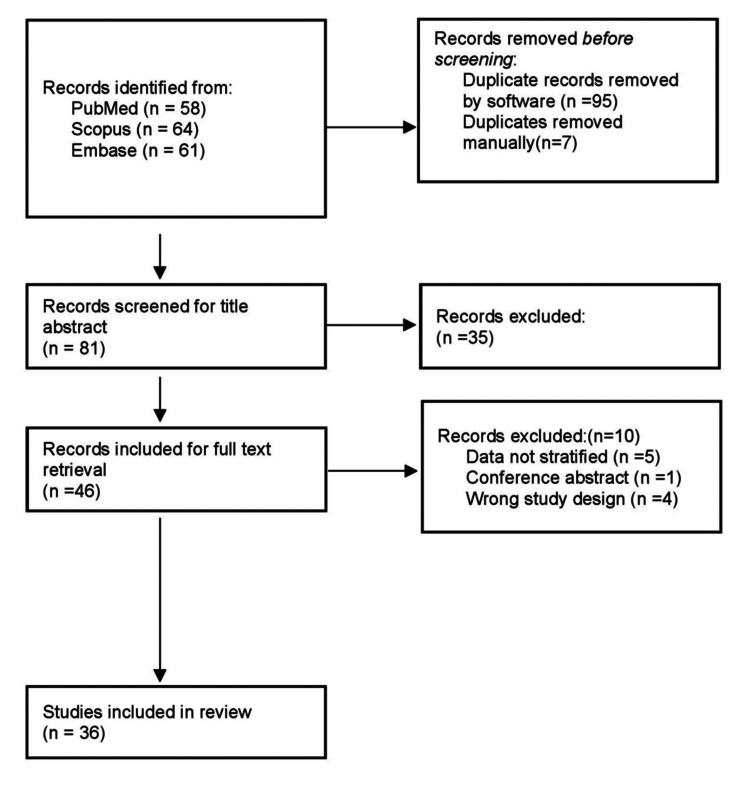
PRISMA flow diagram PRISMA: Preferred Reporting Items for Systematic Review and Meta-Analyses

Figure 1 shows the PRISMA diagram that illustrates the research selection framework. After eliminating 102 duplicate records and examining titles and abstracts, 46 of the 183 items we found in three databases during our literature search were suitable for full-text screening. This systematic review and meta-analysis finally included 36 studies in total.

**Figure 2:**
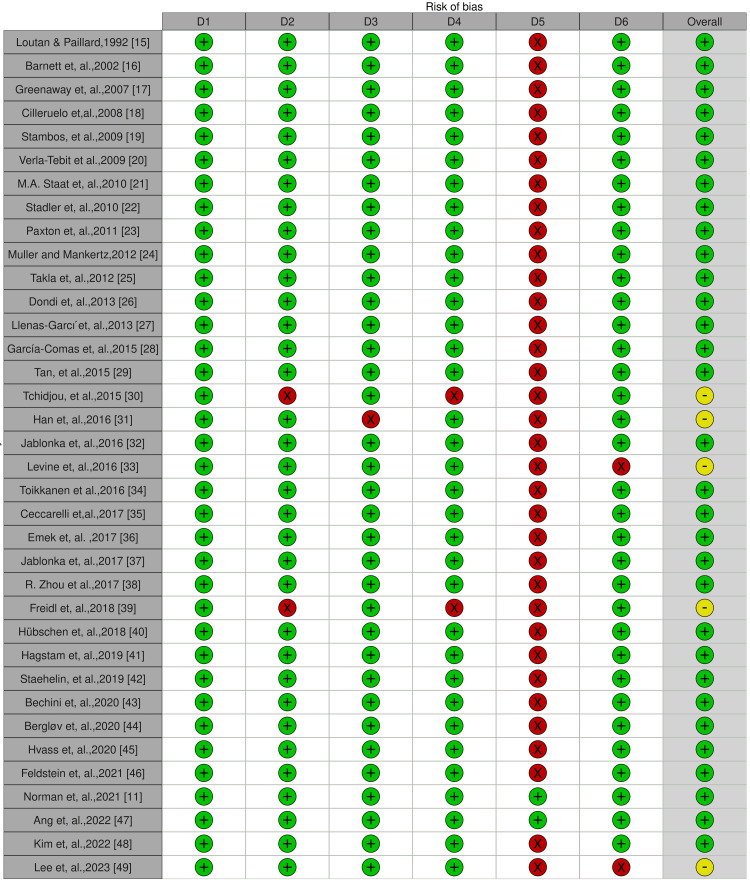
Study quality assessment (risk of bias) chart Judgement: Red: High, Yellow: Some concern, Green: Low D1: Was the research question or objective in this paper clearly stated?; D2: Was the study population clearly specified and defined?; D3: Was the participation rate of eligible persons at least 50%?; D4: Were all the subjects selected or recruited from the same or similar populations? Were inclusion and exclusion criteria for being in the study pre-specified and applied uniformly to all participants?; D5: Was a sample size justification, power description, or variance and effect estimates provided?; D6: Were the outcome measures (dependent variables) clearly defined, valid, reliable, and implemented consistently across all study participants? Reference: [11,15-49]

Figure 2 displays the quality assessment chart for the chosen studies.

Table 1 provides a summary of the features of the studies [11,15-49] that were part of the meta-analysis, such as the author, year, country of origin, population group, and seroprevalence. The studies included were carried out in 17 different nations, with the majority coming from North America (United States and Canada), China, and Europe (Italy, Spain, Germany, Denmark, etc.).

**Table 1:** Seroprevalence of measles antibody among migrant population in different studies NA: not available

Author, year	Host country	Study design	Study setting	Study population group	Age group	Event (total seropositive), n	Sample size, n
Loutan and Paillard,1992 [15]	Nigeria	Cross-sectional study	Community based	Nomadic	NA	186	322
Barnett et al., 2002 [16]	United States	Cross-sectional study	Facility based	Refugee	0–20 years	549	669
Greenaway et al., 2007 [17]	Canada	Cross-sectional study	Facility based	Immigrant/Refugees	Adults	947	1480
Cilleruelo et al., 2008 [18]	Spain	Cross-sectional study	Facility based	Internationally adopted	Children	503	637
Stambos et al., 2009 [19]	United States	Cross-sectional study	Facility based	Foreign-born	17-51 years	171	210
Verla-Tebit et al.,2009 [20]	United States	Cross-sectional study	Facility based	Internationally adopted	Children	117	186
Staat et al., 2010 [21]	United States	Cross-sectional study	Facility based	Internationally adopted	Children	321	382
Stadler et al., 2010 [22]	United States	Cross-sectional study	Facility based	Internationally adopted	Children	185	225
Paxton et al., 2011 [23]	Australia	Cross-sectional study	Facility based	Immigrants	0-17 years	104	115
Poethko-Müller and Mankertz, 2012 [24]	Germany	Cross-sectional study	Community-based	Foreign-born	0-17 years	297	383
Takla et al., 2012 [25]	Germany	Cross-sectional study	Community-based	Asylum seekers	NA	261	300
Dondi et al., 2013 [26]	Italy	Cross-sectional study	Facility based	Internationally adopted	Children	68	85
Llenas-Garcı ´et al., 2013 [27]	Spain	Cross-sectional study	Facility based	Immigrants	>18 years	224	243
García-Comas et al., 2015 [28]	Spain	Cross-sectional study	Community-based	Immigrants	2 - 60 years	1326	1374
Tan et al., 2015 [29]	Singapore	Cross-sectional study	Facility based	Foreign-born	Adults	138	147
Tchidjou et al., 2015 [30]	Italy	Cross-sectional study	Facility based	Internationally adopted	Children	49	67
Han et al., 2016 [31]	China	Cross-sectional study	Community-based	Immigrants	<1 year	148	282
Jablonka et al., 2016 [32]	Germany	Cross-sectional study	Facility based	Refugee	NA	628	678
Levine et al., 2016 [33]	Israel	Cross-sectional study	Community-based	Foreign-born	NA	66	81
Toikkanen et al., 2016 [34]	Germany	Cross-sectional study	Facility based	Asylum seekers	>12 years	18,896	23,647
Ceccarelli et al., 2017 [35]	Italy	Cross-sectional study	Facility based	Migrants	NA	218	256
Emek et al., 2017 [36]	Iran	Cross-sectional study	Community-based	Immigrants	NA	142	182
Jablonka et al., 2017 [37]	Germany	Cohort study	Community-based	Refugee	>12 years	496	552
Zhou et al., 2017 [38]	China	Cross-sectional study	Facility based	Immigrants	NA	256	300
Freidl et al., 2018 [39]	Netherland	Cross-sectional study	Facility based	Asylum seekers	NA	546	620
Hübschen et al., 2018 [40]	Luxembourg	Cross-sectional study	Community-based	Refugee	>13 years	295	406
Hagstam et al., 2019 [41]	Sweeden	Cross-sectional study	Facility based	Immigrants	Adults	1481	1909
Staehelin et al., 2019 [42]	Switzerland	Cross-sectional study	Facility based	Asylum seekers	16-61 years	96	126
Bechini et al., 2020 [43]	Italy	Cross-sectional study	Facility based	Internationally adopted	Children	1213	1870
Bergløv et al., 2020 [44]	Denmark	Cross-sectional study	Facility based	Immigrants	NA	163	177
Hvass et al., 2020 [45]	Denmark	Cross-sectional study	Community-based	Refugee	NA	435	513
Feldstein et al., 2021 [46]	Bangladesh	Cross-sectional study	Community-based	Refugee	NA	887	930
Norman et al., 2021 [11]	Spain	Cross-sectional study	Facility based	Migrants	NA	367	418
Ang et al., 2022 [47]	Singapore	Cross-sectional study	Community-based	Migrants	20-50 years	2021	2234
Kim et al., 2022 [48]	South Korae	Cross-sectional study	Facility based	Migrants	NA	389	419
Lee et al., 2023 [49]	South Korae	Cross-sectional study	Community-based	Migrants	21-63 years	412	547

Geographical Distribution and Population Characteristics

The studies' populations were varied and represented a range of migrant groups such as refugees, asylum seekers, children adopted from other countries, immigrants, children born outside the country in which the study was conducted, and nomadic communities. While the majority of the studies focused on individuals between the ages of 18 and 60, the age range of participants varied. Children, particularly those arriving in new host countries without recorded immunization histories, were included in a number of studies. Data on 42,972 people were reported overall by the studies, with sample sizes varying from 67 to more than 23,647 in each trial. Eleven studies provided findings exclusively from children (0-18 years) [16,18,20-24,26,30,31,43], six studies reported a mixed age group of adults and children [19,28,34,37,40,42], and six studies reported an adult age group of 18-60 years [17,27,29,41,47,49]; a group of 13 studies did not mention any particular age group [11,15,25,32,33,35,36,38,39,44-46,48]. Four studies only presented findings from mothers and migrant women [19,41,48,49]; however, the majority of the investigations were conducted in a mixed population. Immunity levels were found to be possibly influenced by special characteristics such as chronic health disorders (e.g., people living with HIV [27] and women with chronic hepatitis B [44]).

The majority of the studies were from the United States and Europe as the majority of the migration was to the United States and Europe. Among five studies published in the United States, three studies included 793 internationally adopted children from Russia, China, South Korea, Latin America, Eastern Europe, and Southeast Asia revealing a pooled seroprevalence of 76.3% [20-22]. The other two studies, however, focused on foreign-born female guest workers and refugee children [16,19]. A total of 24,693 asylum seekers who migrated from Middle Eastern and North African countries (Syria, Iran, Iraq, Eritrea, Yemen, Armenia, Algeria, Serbia, Sudan, etc.) and some Asian countries (Afghanistan and India) had a pooled prevalence of 82.78%, according to four studies done in European countries (Germany, Switzerland, and the Netherlands) [25,34,39,42]. Three other studies done in Spain among 2035 adult immigrants from non-specific regions worldwide reported a pooled prevalence of 92.17% [11,27,28].

With a pooled prevalence of 83.65%, the studies in the South Asian region mostly came from South Korea, China, and Singapore, and focused on a mixed adult and child population of 4859 immigrants and refugees from South Asian countries, primarily Bangladesh, India, Indonesia, Myanmar, Thailand, Vietnam, China, the Philippines, and others [29,31,35,38,46,48].

Pooled Seroprevalence

**Figure 3:**
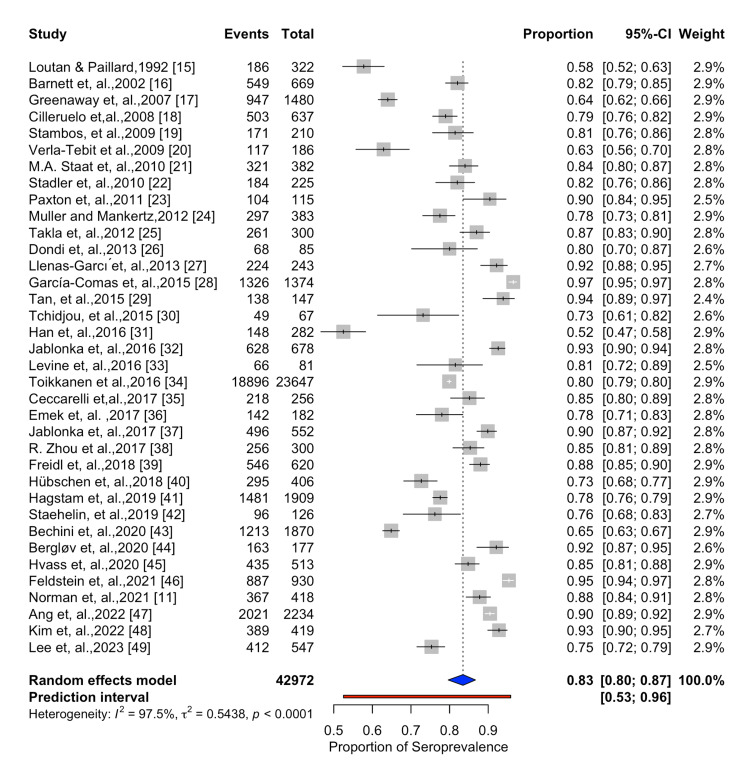
Forest plot showing the estimated measles seroprevalence among migrant population References: [11,15-49]

We calculated the overall pooled seroprevalence of measles antibodies among migrant populations in our meta-analysis. A total of 36 studies were included in the meta-analysis, and the reported seroprevalence data ranged from 52% in China in children aged below one year [31], to 97% in Spain in an immigrant population aged 2-60 years [27]. The seroprevalence estimates across various studies are visually represented in the forest plot (Figure 3), where the size of the boxes represents the weight of each study in the analysis.

The studies' significant heterogeneity (I^2^ = 98%, p < 0.01) suggests that the study population and techniques varied in ways that cannot be entirely accounted for by chance. With a 95% confidence interval (CI) of 80% to 87%, the pooled seroprevalence calculated using the random-effects model was 83%. Significant between-study heterogeneity was indicated by the fact that the prediction interval, which was computed to estimate the range within which the true effect size of a new study might fall, was noticeably wider than the confidence interval of the pooled estimate.

Publication Bias

**Figure 4:**
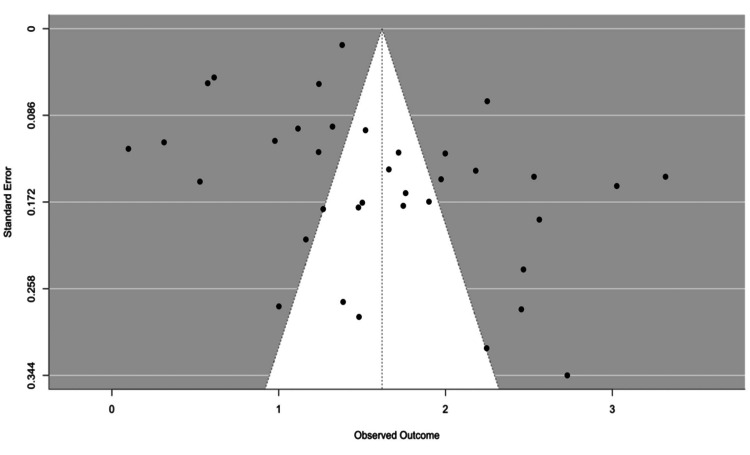
Funnel plot of publication bias related to measles seroprevalence in migrant population

To look for any statistically significant publication bias, we employed Begg's and Eggar's tests. The possibility of publication bias indicates that the small studies have reported considerably lower seroprevalence than the larger studies, according to Eggar's test (p-value<0.05). However, there was no discernible publication bias in the Begg's test result (p-value=0.171). Given this, the significant Egger's test result raises the possibility that our meta-analysis contains some publication bias. The non-significant Begg's test result, however, suggests that any bias of this kind may not be significant or constant among the included studies. The funnel plot shown in Figure 4 supports this conclusion. The funnel plot appears to be asymmetrical, with a slight clustering of studies on the left side. This might indicate a potential for publication bias, where smaller studies with larger effect sizes (to the left) may be more likely to be published.

Meta-Regression

**Figure 5:**
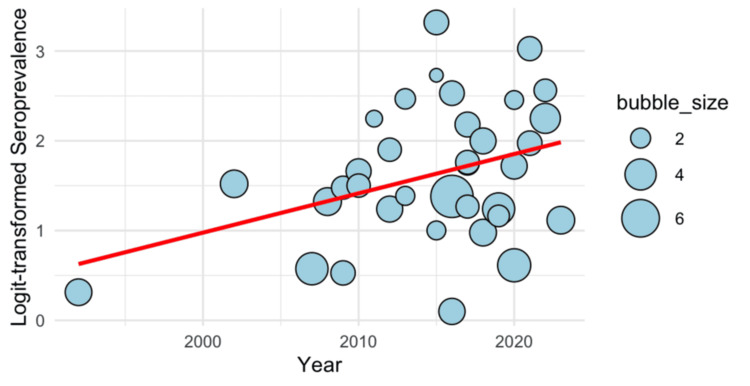
Bubble plot for meta-regression showing time-trend of measles seroprevalence among migrant population over time The circles illustrate the estimates from each study, sized proportionately to the precision of each estimate. A total of 36 observations from 36 studies was considered

In Figure 5, the meta-regression is displayed as a bubble plot. The limited maximum likelihood (REML) approach was utilized to assess between-study heterogeneity, and the model comprised 36 trials (k = 36). Tau (the square root of tau²) was 0.7379. The estimated between-study variance (tau²) was 0.5445 (SE = 0.1372). With an I^2^ value of 98.49%, the model demonstrated a significant degree of heterogeneity, suggesting that heterogeneity between studies, not sampling error, was primarily responsible for the overall range in the effect sizes. The ratio of total variability to sampling variability was represented by the H^2^ value, which was 66.02. Significant heterogeneity among studies was found using a Q-test for heterogeneity (Q (35) =1424.73, p < 0.0001). A statistically significant influence of time on logit-transformed seroprevalence was shown by the meta-regression model results, with an estimated coefficient of 1.6196 (SE = 0.1263, z = 12.82, p < 0.0001). This estimate's 95% confidence interval was between 1.3720 and 1.8672. This implies a positive association between year and seroprevalence over time, with the logit-transformed seroprevalence increasing considerably for each extra year.

Discussion

The results of this systematic review and meta-analysis indicate that there are notable differences depending on geography, demographic subgroups, and context, with an overall pooled seroprevalence of 83% for measles antibodies among migrant populations. Because it highlights the existence of immunity gaps that can pose a risk for outbreaks, particularly in vulnerable categories like children and younger migrants, this seroprevalence, while reflecting a reasonable level of immunity, is nevertheless below the threshold needed to prevent significant outbreaks.

According to the findings, one of the most important factors affecting measles seroprevalence is the nation or region of origin of migrants. Ceccarelli et al. discovered in an Italian study that immigrants from areas with lower vaccination rates, such as Southeast Asia and Sub-Saharan Africa, typically had lower seroprevalence levels when they arrived in their new countries [35]. Due to the importation of cases, the dominant measles strain genotype has occasionally changed dramatically, with new genotypes replacing older ones [50]. This phenomenon is significantly influenced by disparities in vaccination coverage [51].

Despite highly reported coverage, measles transmission has persisted in underdeveloped nations due to vaccination failure caused by variables such as low seroconversion, doubtful vaccine efficacy from cold chain problems, and decreasing immunity [52]. The observed epidemiological diversity is also explained by variations in vaccination programs in different parts of the world, particularly in terms of when they are implemented and how widely they are covered [53]. The current systematic research also found significant disparities between different migration subpopulations. Vaccination gaps frequently disproportionately affect vulnerable groups, including immigrants and asylum seekers. Seroprevalence is significantly influenced by access to medical treatment, especially immunization. Due to socioeconomic circumstances, language challenges, and legal status, migrants frequently encounter obstacles while trying to obtain basic vaccinations. Numerous factors, such as irregular vaccination schedules, limited access to healthcare in host nations, and variations in migratory patterns, can be blamed for this [43].

According to Gogoi et al., serological testing identified immunity gaps not visible from vaccination records alone, and self-reported vaccination status among migrants in the United Kingdom was frequently a poor predictor of real immunity [54]. Measles seroprevalence among immigrant populations might also be influenced by cultural perspectives on vaccinations. Vaccine hesitancy, which reduces vaccination rates, is caused in certain communities by outdated attitudes and false information regarding vaccines. Heywood points out that some migrant groups in Europe have cultural views about vaccines that have led to a decreased uptake of the MMR vaccine, which raises the likelihood of outbreaks, especially among adult migrants who could have missed their childhood immunizations [55]. Social networks are crucial in some immigrant groups when it comes to making decisions about healthcare and immunizations. Influential people or community leaders may have an impact on the group's perception of the value of immunizations. According to Rossi et al., societal norms and collective community views among migrant groups from East Asia and Sub-Saharan Africa affected how they sought medical attention, which led to differing levels of immunity and postponed immunizations when they arrived in their new countries [56].

Due to inadequate immunizations or declining immunity over time, younger migrants typically have lower immunity levels, making age a significant factor in measles seroprevalence among migrant groups. On the other hand, seroprevalence rates are typically greater among older migrants, especially those who have been exposed to natural diseases or who have finished their immunization schedules. Targeted vaccination efforts are necessary to address these age-related discrepancies, especially for younger migrants and those in reproductive age groups, in order to maintain adequate immunity levels and stop outbreaks [36,57,58]. Measles seroprevalence among migrant communities is also strongly impacted by geopolitical events and the movement trends that follow, especially in areas that are undergoing mass migration, displacement, or conflict. According to Yaméogo et al., migration from low-vaccination areas, often brought on by protracted conflicts or inadequate healthcare systems, causes immunity gaps and impairs the effectiveness of vaccination campaigns in nearby regions [59]. Similarly, the inflow of refugees from areas with inadequate healthcare systems during the 2015 European migrant crisis in Germany and the current Syrian refugee crisis in Turkey resulted in varying measles seroprevalence levels in the host countries [37,59]. These immunity disparities are also made worse by policy-level variables, such as the absence of uniform vaccination recommendations for migrants.

It is important to take into account the many limitations of this study. First, bias may be introduced and the findings' capacity to be applied broadly may be limited by regional variations in study methods, sample sizes, and populations. Furthermore, because vaccination laws and migration patterns change over time, the included studies' geographic and chronological breadth might not accurately represent contemporary trends. Second, there was a lack of information on certain sensitive groups, such as children and illegal immigrants, which could have masked significant variations in immunity levels. Another potential limitation is the possibility of publication bias, where studies with significant or noteworthy findings are more likely to be published, potentially skewing the overall results by overrepresenting such studies. Lastly, our knowledge of the epidemiological consequences of genotype alterations and their effects on immunity and outbreak risk in migrant communities is limited by the included studies' lack of comprehensive data on measles genotypes. Also, this review’s reliance on observational studies introduces certain methodological constraints, as these designs primarily capture associations rather than causation, which may affect the strength of inferences about seroprevalence levels and immunity trends. 

This review and meta-analysis show the considerable variation in measles seroprevalence among migrant groups in various contexts by combining data from all across the world over a number of years. The high heterogeneity (I² = 98%) likely stems from variations in study settings (e.g., community-based vs. facility-based), age groups (e.g., children vs. adults with differing immunity levels), and geographic origins (reflecting diverse immunization rates), each influencing measles immunity levels across migrant subgroups and highlighting the need for subgroup-specific analysis in future research. It emphasizes that, in many migrant subpopulations, the overall seroprevalence is still below the essential threshold needed for herd immunity, even in the face of international attempts to increase vaccination coverage. In order to address these differences and stop any outbreaks, more focused public health measures are required. This immunity gap has remained throughout time, particularly in vulnerable groups including refugees, asylum seekers, and younger migrants.

## Conclusions

Measles remains a major public health concern despite notable advancements in increasing vaccine coverage and lowering overall incidence, morbidity, and mortality, especially among migratory groups where immunity gaps are still prevalent. Due to their increased risk of measles complications, vulnerable populations like children, refugees, and asylum seekers are most affected by these gaps. Particularly among younger migrants and those from areas with historically lower vaccination rates, the current worldwide measles seroprevalence among migrant populations is still below the threshold needed to establish herd immunity.

Coordinated public health initiatives are desperately needed to increase vaccine coverage, especially among migrant communities, given the ongoing immunity gaps. Achieving sufficient protection against measles requires targeted vaccination efforts that target high-risk categories, women of reproductive age, and younger migrants. In order to reduce the likelihood of future outbreaks and guarantee that all people are sufficiently protected from this extremely contagious disease, it will be imperative to strengthen these efforts.
